# Conference Report: ACCURACY IN POWDER DIFFRACTION II: A REPORT ON THE SECOND INTERNATIONAL CONFERENCE Gaithersburg, MD May 26–29, 1992

**DOI:** 10.6028/jres.098.019

**Published:** 1993

**Authors:** Judith K. Stalick

**Affiliations:** Reactor Radiation Division, National Institute of Standards and Technology, Gaithersburg, MD 20899-0001

## 1. Introduction

The purpose of this conference was to assess the progress that has been made in the theory, techniques, and applications of powder diffraction data since the first highly successful symposium on Accuracy in Powder Diffraction held at NIST (then the National Bureau of Standards) in 1979, as well as to define problem areas where further work is needed. The proceedings of the first symposium, published as *Accuracy in Powder Diffraction* (NBS Special Publication 567), have been widely cited in the literature, testifying to their importance as resources for active researchers in powder diffraction. However, the 13 intervening years have seen further major advances in the practice of this technique.

This conference was organized by the Commission on Powder Diffraction of the International Union of Crystallography (IUCr), and was sponsored jointly by NIST, the IUCr, and the JCPDS-International Centre for Diffraction Data (ICDD). The meeting was attended by 177 registrants, representing 18 countries and five continents. The international program committee, chaired by R. J. Hill (CSIRO, Australia), selected the 26 invited speakers, and another 11 oral presentations were chosen from the 70 contributed abstracts. The program was divided into six topics: phase identification and quantification; accuracy and standards; new developments in software and data analysis; profile fitting, decomposition, and microstructural effects; novel applications and structural science; and new developments in hardware, including detectors, and studies under non-ambient and time-resolved conditions. The program for the last session was designed to interface smoothly with the Workshop on X-Ray and Neutron Diffraction at High Pressure, organized by the High Pressure Group of the IUCr, which was held immediately after the conference at the Geophysical Laboratory of the Carnegie Institution in Washington, DC. In addition, two tutorial workshops, organized by the ICDD, were conducted as part of the conference, one on powder diffractometer sensitivity and one on indexing methods.

The sessions opened with a tribute to the late William Parrish detailing his contributions to powder diffraction, delivered by T. C. Huang of IBM. Bill Parrish was responsible for developing many of the instruments and analytical methods that are used for powder diffraction analysis; his pioneering efforts will make possible even further advances.

## 2. Phase Identification and Quantification

The first keynote presentation by B. L. Davis (South Dakota School of Mines and Technology) discussed the development and application of quantitative phase analysis with reference intensity ratios (RIR). He included in his presentation helpful experimental techniques as well as applications to mineralogical samples. L. Zevin (Ben-Gurion University, Israel) then discussed standardless methods of phase analysis, in which a system of *n* phases can be determined provided that at least *n* mixtures of these phases are available. In the second keynote talk, R. L. Snyder (Alfred University) led the participants through the mathematical relationships developing a consistent set of notation for each of the RIR-based methods for quantitative analysis, including the standardless methods as well as the whole-pattern fitting and Rietveld techniques. J. K. Stalick (NIST) then discussed phase analysis with the Rietveld method in further detail; using neutron diffraction data, minor impurity phases were quantitatively determined even when there were no apparent peaks observable. A statistical approach to microabsorption corrections was presented by H. Hermann (Institute of Solid State Research, Dresden, Germany), based upon particle-size distributions and surface roughness. M. S. Nakhmanson (Comphys Lab Enterprise, St. Petersburg, Russia) discussed theory and techniques for phase identification using the PDF database. The organization of the database into clusters of materials with similar structure can result in broadened as well as more efficient database uses. Additional presentations were made by R. G. Marquart (Fein-Marquart Associates) on the user-assisted autocalibration process in the (μPDSM micro powder diffraction search/match, D. K. Smith (Penn State University) on the limiting effect of crystallite size on accuracy in quantitative powder diffraction analyses, and H. Oettel (Technical University of Freiberg, Germany) on x-ray phase analysis in textured materials.

## 3. Accuracy and Standards

In the opening address for this session, P.-E. Werner (University of Stockholm, Sweden) discussed the relative merits of cameras and diffractometers for x-ray diffraction measurements, presenting detailed experimental comparisons. Although the diffractometer is clearly to be preferred for accurate intensity measurements, the high angular resolution of a Guinier camera results in very accurate position measurements. J. F. Bérar focused on data optimization and propagation of errors, with particular application to the Rietveld method using x-ray data. He showed that the details of peak shapes and sample effects can result in systematic errors. J. P. Cline (NIST) described the characterization and use of XRD standard reference materials, and H. Toraya (Nagoya Institute of Technology, Japan) discussed the accurate determination of unit-cell parameters using an internal standard reference material and whole-pattern fitting.

## 4. New Developments in Software and Data Analysis

The exciting emergence of techniques for *ab initio* structure solution from powder data was the focus of two keynote talks and an additional oral presentation. L. B. McCusker (Institute of Crystallography, ETH-Zentrum, Switzerland) reviewed the available software, and presented some examples of structures solved from powder data. She emphasized the importance of minimizing the degree of peak overlap by improving sample crystallinity and instrumental resolution. J. M. Newsam (Biosym Technologies Inc.) described direct space methods of structure solution, utilizing model-building methods coupled with interactive simulation of data with comparison against experiment. The simulated annealing method has been successfully applied to 90% of known zeolite structures. M. Estermann (Institute of Crystallography, ETH-Zentrum, Switzerland) gave details of the fast iterative Patterson squaring (FIPS) method, an improved treatment of severely overlapping reflections in a powder pattern for the application of direct methods. The third keynote talk of this session was a review of the procedures and applications of automatic indexing by D. Louër (Université de Rennes, France). He stressed the importance of high precision in the measurement of peak positions, and surveyed the different computer-based approaches. B. H. Toby (Air Products and Chemicals, Inc.) discussed recent advances in the determination of atomic pair distribution functions for the analysis of short-range order in crystalline and non-crystalline materials, and J. Schneider (Universität München, Germany) showed how short-range order effects could be combined with standard Rietveld refinement.

## 5. Profile Fitting, Decomposition, and Microstructural Effects

The first keynote presentation of this session by J. I. Langford (University of Birmingham, UK) reviewed the use of the Voigt function in determining microstructural properties from diffraction data by means of pattern decomposition, including all formulas and codes necessary for the analysis. Again, high resolution and good counting statistics are necessary to obtain reliable information. The second keynote address by V. Valvoda (Charles University, Czechoslovakia) discussed both experimental techniques to reduce preferred orientation effects and various methods of correcting the model. Finally, T. M. Holden (Chalk River Laboratories, Canada) reviewed residual stress measurements with neutrons, and A. Le Bail (Université du Maine, France) discussed the current status of modelling anisotropic line-broadening effects in Rietveld analysis.

## 6. Novel Applications and Structural Science

The applications of powder data analysis to structure problems now extend beyond the Rietveld refinement of positional parameters. D. L. Bish (Los Alamos National Laboratory) described in a keynote address how the traditional Rietveld method could be applied to structure solution when used, for example, in conjunction with distance least-squares constraints and crystal-chemical principles or with high-resolution TEM data. Several examples of zeolites and clay mineral structures were given. H. Boysen (Universität München, Germany) showed how anharmonic thermal parameters could be determined using the Rietveld method, with examples of diffusion paths in fast-ion conductors. A high resolution synchrotron x-ray powder diffraction and molecular modelling study of the structural changes accompanying absorption of trans-stilbene into H-ZSM-5 zeolite was described by J. B. Parise (SUNY at Stony Brook). The second keynote talk by J. P. Attfield (University of Cambridge, UK) reviewed recent advances in the use of anomalous dispersion in powder diffraction studies, including the use of synchrotron x-ray wavelengths that are close to an elemental absorption edge. He showed that using this technique one could determine cation distribution and oxidation states. R. W. Cheary (University of Technology, Sydney, Australia) then discussed how line broadening effects could be employed to study inter-growths and antiphase domains in barium hollandite compounds, and R. Vargas (IVIC, Caracas, Venezuela) described a physical model for x-ray analysis of order-disorder in lamellae of myelinated nerve sheaths.

## 7. New Developments in Hardware, Including Detectors, and Studies Under Non-Ambient and Time-Resolved Conditions

In the keynote address of this session, Y. Fujii (University of Tokyo, Japan) reviewed the utility of the diamond-anvil cell for investigations of powders under high pressure using synchrotron x-ray radiation, and described the high-pressure facilities available at the Photon Factory in Tsukuba. Of particular interest was the use of imaging plates to provide data suitable for Rietveld refinement. Synchrotron radiation was also used by L. W. Finger (Carnegie Institution of Washington) in the study of sub-micron single crystals of bismuth, and by M. Sutton (McGill University, Canada) for time-resolved x-ray diffraction studies of crystallization in metallic glasses. The high temperature x-ray determination of phase transitions in large grain alloys was discussed by O. B. Cavin (Oak Ridge National Laboratory). J. S. Loveday (Rutherford Appleton Laboratory, UK) reviewed two important new developments in the field of neutron diffraction at high pressure: a large-volume cell which allows Rietveld refinement above pressures of 10 GPa, and the development of a new detector at a Russian reactor. J. A. Fernandez-Baca (Oak Ridge National Laboratory) described the new multidetector powder diffractometer at Oak Ridge. Finally, crystal structure analysis by pattern fitting of energy-dispersive powder diffraction data, using synchrotron radiation at high pressure, was described by T. Yamanaka (Osaka University, Japan).

## 8. Summary

This conference made it quite clear that the renaissance in powder diffraction has continued at an even greater pace since the first meeting on Accuracy in Powder Diffraction in 1979. The variety and quality of information that can be extracted from a powder pattern has greatly increased, particularly with the application of whole-pattern or Rietveld methods; the ability to study materials under non-ambient conditions has also been expanded. In a summary statement, R. J. Hill and D. E. Cox have stated, “Diffraction patterns previously collected only for phase identification or unit cell determination are now used to study such things as the subtleties of electron density distribution, the mechanism of phase transformations, and the distribution of cation oxidation states. We can look forward eagerly to APD-III, but perhaps we should recognize the pace of change is now so great that we should not wait another 13 years for this!”

Proceedings from this conference have been published as NIST Special Publication 846. You may contact either of the conference co-chairmen (J. K. Stalick and E. Prince, both of the Reactor Radiation Division) for further information.

